# Supplementary material for: Comparative multiomics analysis of cell physiological state after culture in a basket bioreactor
Source: Sci Rep. 2022 Nov 23;12:20161. doi: 10.1038/s41598-022-24687-4 (PMC9686226; doi:10.1038/s41598-022-24687-4)
Supplement: Supplementary file 1 — Supplementary Information 1. [file 41598_2022_24687_MOESM1_ESM.zip › raw data/Transcriptomic raw data/7.AS/2.dasplot/TE1vsTC1/A5SS/2_VPS29_NW_023666037.1_139383463_139383474_+@NW_023666037.1_139383463_139383552_+@NW_023666037.1_139386392_139386583_+.pdf]

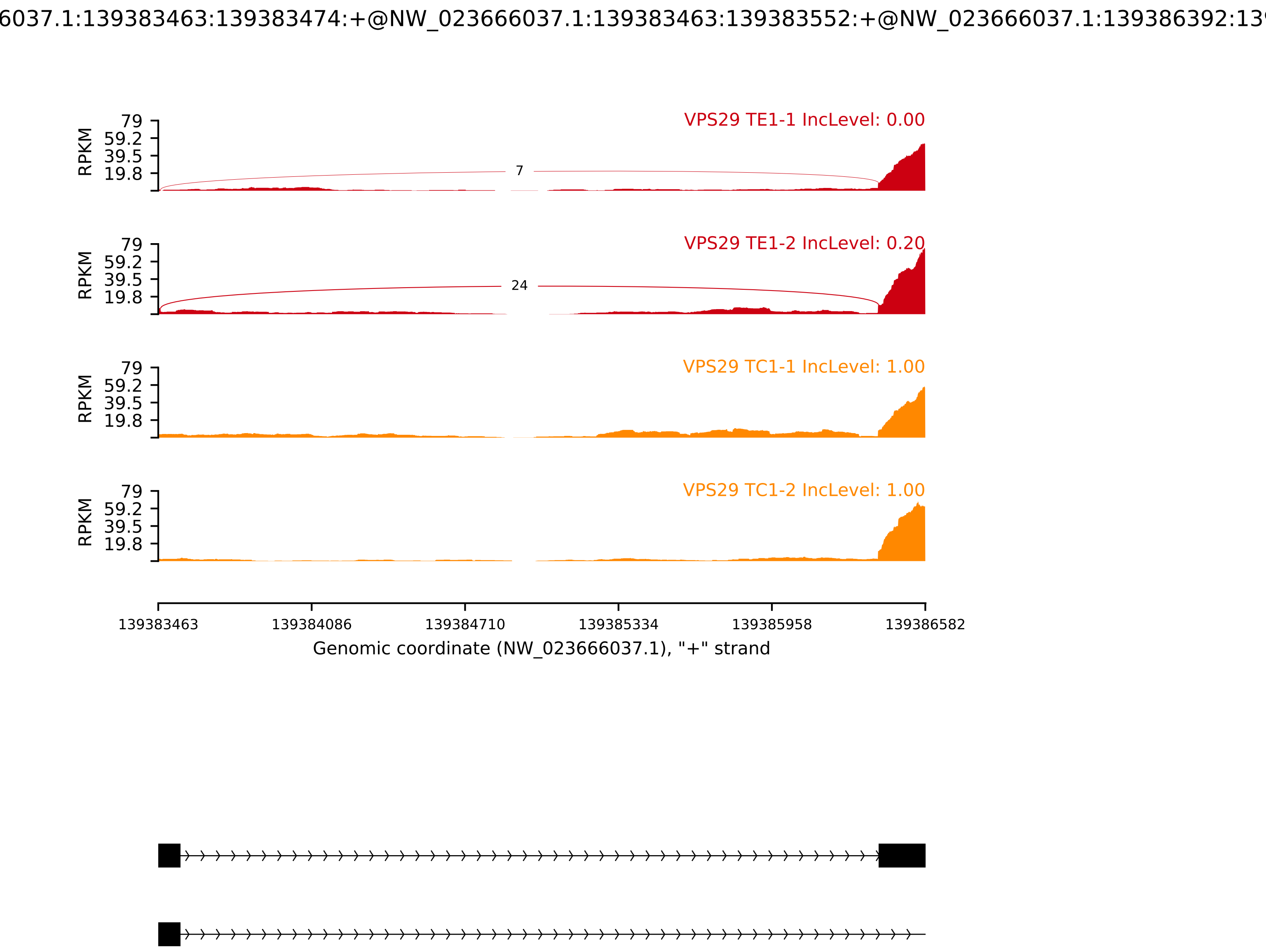
6037.1:139383463:139383474:+@NW_023666037.1:139383463:139383552:+@NW_023666037.1:139386392:13
VPS29 TE1-1 IncLevel: 0.00
VPS29 TE1-2 IncLevel: 0.20
VPS29 TC1-1 IncLevel: 1.00
VPS29 TC1-2 IncLevel: 1.00
RPKM
79
59.2
39.5
19.8
7
24
139383463
139384086
139384710
139385334
139385958
139386582
Genomic coordinate (NW_023666037.1), "+" strand
